# The Quest for Immunity: Exploring Human Herpesviruses as Vaccine Vectors

**DOI:** 10.3390/ijms242216112

**Published:** 2023-11-09

**Authors:** Mohamed S. Kamel, Rachel A. Munds, Mohit S. Verma

**Affiliations:** 1 Department of Agricultural and Biological Engineering, Purdue University, West Lafayette, IN 47907, USA; 2 Department of Medicine and Infectious Diseases, Faculty of Veterinary Medicine, Cairo University, Giza 11221, Egypt; 3 Krishi Inc., West Lafayette, IN 47906, USA; 4 Weldon School of Biomedical Engineering, Purdue University, West Lafayette, IN 47907, USA; 5 Birck Nanotechnology Center, Purdue University, West Lafayette, IN 47907, USA

**Keywords:** *Herpesviridae*, recombinant vaccines, vaccine vectors, HSV, VZV, CMV

## Abstract

Herpesviruses are large DNA viruses that have long been used as powerful gene therapy tools. In recent years, the ability of herpesviruses to stimulate both innate and adaptive immune responses has led to their transition to various applications as vaccine vectors. This vaccinology branch is growing at an unprecedented and accelerated rate. To date, human herpesvirus-based vectors have been used in vaccines to combat a variety of infectious agents, including the Ebola virus, foot and mouth disease virus, and human immunodeficiency viruses. Additionally, these vectors are being tested as potential vaccines for cancer-associated antigens. Thanks to advances in recombinant DNA technology, immunology, and genomics, numerous steps in vaccine development have been greatly improved. A better understanding of herpesvirus biology and the interactions between these viruses and the host cells will undoubtedly foster the use of herpesvirus-based vaccine vectors in clinical settings. To overcome the existing drawbacks of these vectors, ongoing research is needed to further advance our knowledge of herpesvirus biology and to develop safer and more effective vaccine vectors. Advanced molecular virology and cell biology techniques must be used to better understand the mechanisms by which herpesviruses manipulate host cells and how viral gene expression is regulated during infection. In this review, we cover the underlying molecular structure of herpesviruses and the strategies used to engineer their genomes to optimize capacity and efficacy as vaccine vectors. Also, we assess the available data on the successful application of herpesvirus-based vaccines for combating diseases such as viral infections and the potential drawbacks and alternative approaches to surmount them.

## 1. Introduction

Vaccination is one of the essential strategies for preventing and controlling serious infections in humans and animals. Most of the commercially available vaccines are either first- or second-generation vaccines. While the first-generation vaccines comprise inactivated and live attenuated vaccines, so-called second-generation vaccines also include subunit vaccines, DNA vaccines, gene-deleted vaccines, and recombinant vector vaccines [1]. Although inactivated vaccines induce a satisfactory humoral immune response and are more commonly applied to date, they (i) need a potent adjuvant, (ii) primarily only trigger short and relatively weak humoral immune responses, (iii) can require high biosecurity level production facilities, and (iv) require large volume inocula as they do not replicate inside the host. Meanwhile, although live attenuated vaccines provoke a long-lasting, cell-mediated, and humoral immune response, reversion to virulence or lack of safety in immunocompromised individuals constitute problematic issues. Drawbacks of other vaccine approaches have been recently reviewed and include low immunogenicity, the risk of genomic integration, or the requirement of low-temperature storage and transportation [2,3,4,5].

Viral vectors are promising tools for developing new vaccines and immunization strategies [6,7,8]. Viral vector-based vaccines possess merits over conventional vaccines as they retain immunogenicity without using adjuvants and cause a robust cytotoxic T lymphocyte (CTL) response [6]. Within the last decades, viral vectors have been extensively examined in animals, and some viruses have even been produced as vaccine vectors and entered clinical trials [7,9,10,11]. Here, vaccination protocols often require “prime-boost” schemes with different vaccine categories to enhance the immune response to a particular pathogen [12,13], and common viral vectors include adenoviruses, lentiviruses, poxviruses, and herpesviruses [14,15].

**Figure 1:**
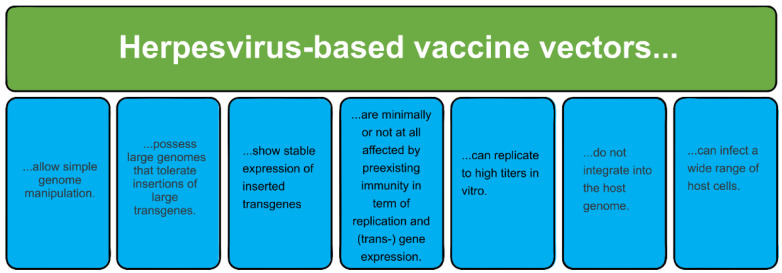
Advantages of the use of human herpesviruses (HSVs, CMV, VZV) as recombinant vaccines.

Of the aforementioned viral vectors, herpesviral vectors have also been applied in vaccine development targeting human pathogens and cancer. The eight herpesviruses that infect humans (human herpesviruses; HHVs) include herpes simplex virus 1 (HSV-1, or HHV-1), herpes simplex virus 2 (HSV-2, or HHV-2), Varicella Zoster virus (VZV, or HHV-3), Epstein–Barr virus (EBV, or HHV-4), human cytomegalovirus (HCMV, or HHV-5), HHV-6, HHV-7, and Kaposi Sarcoma herpesvirus (KSHV, or HHV-8) [16,17,18]. Herpesviruses are enveloped viruses with a large linear double-stranded DNA genome with many open reading frames (ORFs) [19], which serve as ideal insertion sites for foreign DNA fragments. Most herpesviruses can infect various cell types except for germinal cells of the reproductive system. They tightly regulate their canonical gene expression with immediate-early (IE) genes, followed by early (E) and late (L) genes. While most IE genes are responsible for the production of regulatory proteins, early gene expression leads to enzyme production [20]. Interestingly, not all IE genes encode regulatory genes. While *ICP4*, *27*, *22*, and *0* encode regulatory products that function during transcription, RNA processing, and translation; the IE-infected cell protein 47 (ICP47) encodes a gene product that interferes with the transporter associated with antigen processing (TAP)-mediated antigen presentation [21]. Late genes usually encode all structural proteins [20,22]. In the case of HSV-1, which is probably the most studied HHV, lytic infections can result in the lysis of infected cells except when the virus infects neurons of the peripheral nervous system (PNS), where, instead, it enters a “latent” or quiescent state in which the viral genome is silenced except for the expression of the latency-associated transcripts (LATs). Reactivation of latent viruses can again lead to the induction of the lytic replication cycle where progeny virions are transported back to the site of primary infection and induce an immune response, leading to clinical symptoms and resulting in the shedding of the virus and spread to other individuals [23,24,25]. The viruses discussed here possess many desirable properties that make them perfect choices as viral vectors (Figure 1).

Recent advances in molecular biology have enabled the development of HHV-based vaccine vectors. Most importantly, the bacterial artificial chromosome (BAC) technology facilitated the easy genetic manipulation of the large HHV genomes straightforwardly [8,16,26], and a technique known as en passant mutagenesis greatly simplified the insertion of foreign genes into HHV genomes, the deletion of non-essential genes, as well as insertion of point mutations [27,28,29]. Additionally, codon optimization could enhance the expression of inserted transgenes of interest in specific cell types [30,31].

Indeed, herpesvirus-based vectors are not only evaluated as vaccine vectors against several infectious diseases but also for brain tumors as immunotherapy and as a gene delivery vector to the central nervous system (CNS) due to their cell tropism [32,33]. It is their promise as ideal vaccine vectors that has led us to this review. In this review, we discuss the underlying molecular structure of herpesviruses, how they are being developed for vaccine vectors, and their successful applications as vaccines. In conducting this review, we used PubMed, Web of Science, and Google Scholar to collect and extract relevant studies. The literature searches were conducted without limiting the time period to ensure a comprehensive evaluation of the available literature. The keywords used in the search included but were not limited to “human herpesvirus vector vaccines”, “herpesvirus-based vector vaccines”, “human herpesvirus vector vaccines immunity”, “human herpesviruses vector vaccines immune response”, as well as individual human herpesviruses such as HSV, along with terms like “viral vector” and “vector vaccine”. By employing these search strategies, we aimed to capture a wide range of studies focusing on herpesvirus-based vector vaccines. This approach allowed us to encompass both recent and earlier studies, providing a comprehensive analysis of the topic.

### 1.1. Herpesvirus Morphology

**Figure 2:**
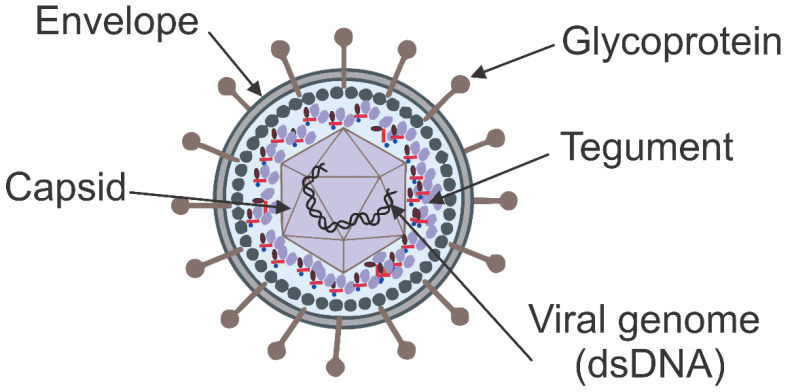
Schematic structure of herpesviruses. This schematic diagram illustrates the complex architecture of herpesviruses composed of four main components. The outer envelope, produced during viral budding, contains viral glycoproteins that facilitate viral attachment, entry, and evasion of host immune response. Inside this envelope is the capsid, an icosahedral structure protecting the viral genome and its associated proteins. The genome, a large double-stranded DNA molecule, carries essential genetic information for viral replication. Depending on the herpesvirus type, this genome can either be linear or circular. Interposed between the capsid and envelope is the tegument layer, essential for viral assembly and gene expression regulation. Notably, herpesviruses’ envelopes comprise numerous glycoproteins enabling viral binding to host cells and facilitating viral envelope fusion with host cell membranes.

Herpesvirus virions are spherical and consist of four main components: the core, the capsid, the tegument, and the envelope (Figure 2). Its diameter depends on the respective viral species and ranges from 120 to 200 nm due to diverse capsid-tegument envelope sizes [34,35]. The herpesvirus core comprises a single linear, double-stranded DNA packaged into the icosahedral capsid. The outer capsid diameter size ranges from 125 to 130 nm and consists of 162 capsomers (150 hexons and 12 pentons), each containing six and five copies of the major capsid protein, respectively. These capsomers are connected through the triplexes, each of which includes two copies of one protein and one copy of another. The tegument, which encompasses the capsid, comprises more than 30 viral protein species and is only inadequately characterized in terms of its structure [34]. The lipid envelope encases the outer tegument, where viral membrane glycoproteins and some cellular proteins are located. The protein composition of the envelope and tegument differs extensively within the *Herpesviridae* [34].

Herpesvirus genomes are not highly conserved. They differ in size, structure, and organization, which reflects the complex taxonomic categorization of *Herpesviridae*. In general, their genomes consist of two unique (non-repetitive) regions, unique short (US) and unique long (UL), that are separated by repeated sequences which occur at both ends of the linear genomes. In some *Herpesviridae* members, these repeated sequences may also occur internally [36]. Two or four different genome isomers that occur in equimolar ratios are generated during virus replication due to inversions of the unique sequences [37,38].

As mentioned above, the genes of herpesviruses can be categorized into three groups of transcripts: IE, E, and L genes. While the first two groups (IE and E) encode proteins with regulatory functions and are required for viral replication, the third group (L) produces structural proteins. Within the three transcriptional classes, non-essential genes were detected in some members of the *Herpesviridae* family. These non-essential genes are dispensable for viral replication and progeny production [20,34,39]. However, some growth-regulating and immunomodulatory proteins and cellular gene homologs have also been shown to be dispensable for the virus to replicate in tissue culture but encode regulatory proteins responsible for modulation of the host immune response and regulation of virus growth [40].

The herpesvirus envelope contains glycoprotein spikes that are involved in the process of binding and entry of the host cell [34]. Most HHV genomes remain as circular episomal molecules during the latent state, with HHV-6 as an intriguing exception [41]. The fact that these viruses do not integrate into the cellular genome is an essential consideration for their use as vectors.

### 1.2. Evolutionary Process of Herpesviruses

The evolutionary journey of herpesviruses is complex, spanning millions of years and involving various stages. It is believed that herpesviruses originated from an ancestor that existed over 400 million years ago [42,43]. This ancient virus likely infected early vertebrates, leading to the diversification and emergence of the different herpesvirus subfamilies we see today. Herpesviruses have coevolved with their host species over extended periods, and this coevolutionary relationship has influenced the genetic composition of both the viruses and their hosts, establishing a delicate balance of viral replication and host immune control. This balance permits herpesviruses to persist in their hosts. As they evolved, herpesviruses expanded their host range by adapting to infect different species. This expansion occurred through mechanisms such as cross-species transmission and host switching events [44,45]. As a result, zoonotic infections emerged where herpesviruses jumped from animals to humans. Throughout their evolutionary history, herpesviruses have acquired and lost genes, which has resulted in their increased capacity to infect an array of host species. The viral genes involved in immune evasion, viral replication, and modulation of host cell functions were primarily acquired through horizontal gene transfer events. Notably, herpesviruses have developed the capacity to create latency infections in their host cells. During latency, the viral genome remains silent and persists in a quiescent state. However, periodic reactivation from latency can occur, leading to viral replication and shedding. This contributes to viral transmission and the spread of infection [46,47]. Yet, more research is needed for a better mechanistic and evolutionary understanding of herpesviruses, as this is critical for a better knowledge of their biology and pathogenesis. Furthermore, it aids in developing effective vaccines and antiviral therapies.

### 1.3. Herpesvirus Biology

Numerous different herpesviruses have been detected in different species, and more are expected to be identified in the future [48]. While herpesviruses are usually host-restricted, human activities that involve close contact with different animal species from such places as zoos, farms, or from our own home by keeping pets can result in the zoonotic transmission of herpesviruses [49]. Some *Alphaherpesvirinae* members, in experimental animal systems, can infect a number of species, whereas *Beta-* and *Gammaherpesvirinae* have a limited host range. Similarly, prevailing utterances characterize their growth in tissue culture [50].

Herpesviruses can remain within healthy hosts for extended times during latency; however, in hosts with weakened immunity, such as the elderly, neonates, and immune-compromised populations, viral infections can be severe and even result in the death of some patients [51,52]. Aerosol spread and mucosal contact are the most natural transmission routes. HSV-1 and HSV-2 are mainly sexually transmitted infections, with most reported new infections infecting the genitals [53]. The systemic infection of most herpesviruses is mainly secured via cell-associated viremia. However, infection with some members of the genus Simplexvirus is confined to the epithelium and innervates sensory neurons at the inoculation site. Herpesviruses have extensive and complicated ways of tempering host responses to infection and can establish permanent latent infections. In clear, general terms, the cell types implicated in latency are lymphocytes (*Gammaherpesvirinae*), monocytes (*Betaherpesvirinae*), and neurons (*Alphaherpesvirinae*) [47,54,55].

### 1.4. Herpesvirus Genomes

Herpesvirus genomes have variable sizes ranging from 124 to 259 kbp, mostly arranged in 70 to 165 genes [56]. Before the vast sequence data generation, genome structures were used to assist in the taxonomy. Nevertheless, the advantage of this criterion is restricted, as comparable structures have emerged more than once in the family. Nucleic acid hybridization data (as serological input) also provided information, but both were restricted to exhibiting relationships between closely related viruses [57]. Data obtained from nucleotides and amino acid sequences have enabled increasing distinction and now predominate herpesvirus classification [56,58,59]. It has been widely considered that herpesviruses have possibly adapted to their hosts over time, indicating that a significant degree of co-evolution has occurred. Similarities between the phylogenetic relationships among herpesviruses and those among their hosts implement substantial support for this model, and in some cases, it is revealed that co-speciation occurs [56,58,59].

### 1.5. Herpesvirus Taxonomy

Herpesviruses are divided into three groups: α herpesviruses (VZV and HSV-1 and -2) with a short-replicated cycle, having a broad host range and inducing cytopathology in monolayer cell cultures; *β* herpesviruses (HHV-6, HHV-7, and CMV) with a long-replicating cycle and limited host range; and γ herpesviruses (HHV-8 and EBV) with a very restricted host range [60,61].

Traditionally, herpesviruses have been classified since 1971 by the International Committee on Taxonomy of Viruses (ICTV) [62]. A temporary strategy to provide herpesviruses with approved names [63] was followed by categorizing them into subfamilies, primarily based on biological criteria [22]. These efforts were somewhat valuable; however, they were not free from a few misclassifications [64]. To improve classifications, the subfamilies were additionally subdivided into genera employing molecular data to a greater extent than before; this principally concerned genome characteristics such as structure and size [56,65]. In the report of the ICTV Herpesvirus Study Group [57,66], the family *Herpesviridae* comprises three subfamilies: *Alphaherpesvirinae* (including the *Simplexvirus*, *Mardivirus*, *Varicellovirus*, and *Iltovirus* genera), *Betaherpesvirinae* (including the *Cytomegalovirus*, *Roseolovirus,* and *Muromegalovirus* genera), and *Gammaherpesvirinae* (including the *Rhadinovirus* and *Lymphocryptovirus* genera). The genus *Ictalurivirus* is not part of any subfamily, and several species are not designated to any genera. Apart from one virus that affects mammals or birds, most viruses belonging to this taxa have lower vertebrate (reptilian, amphibians, and fish) or invertebrate (bivalve) hosts. Finally, a considerable number of unassigned herpesviruses exist [58].

### 1.6. Replication-Competent Herpesvirus Vectors

Replication-competent vectors originate from attenuated viruses via deleting or mutating non-essential genes for growth in cultured cells in vitro. Eliminating one or more unnecessary genes may diminish pathogenicity without requiring a cell line to complement growth [67]. HSVs are promising vectors for numerous purposes in human healthcare, such as the discriminating killing of cancer cells, prevention of infectious diseases, transfer and expression of human genes to nervous systems, and targeting the infection into specific organs or even tissue parenchyma [32,33,68]. Recombinant HSV vectors can also be simply produced without wild-type (wt) contamination at high titers and purity [69,70].

Moreover, the virus latency behavior may be employed for durable long-term therapeutic transgene expression in neurons. Unless the replication-competent virus is somehow compromised, it will still infect and kill the target cells even before the therapeutic genes are actively expressed. Thus, only oncolytic HSV vectors have this desired approach, called the “arming” of the oncolytic virus, to aid in virus replication, enhance cellular immunity, or play a major role in tumor cell death [71,72]. Recombination of a replication-competent vector with a wild-type virus can happen, although this is an extremely rare event and is unlikely to generate progeny that is more pathogenic than wild-type HSV.

Numerous genes implicated in HSV replication, immune evasion, and virulence are unnecessary for the viral life cycle in vivo that have been verified [73]. These genes are usually associated with various interplays and interactions of these related genes to cellular proteins that optimize the virus growth capability in cells. Understanding this interaction has enabled us to delete/modify these genes, alone or in combination, to construct HSV mutants with a diminished capability to reproduce in normal resting cells. However, the resulting virus can replicate in dividing or tumor cells. These viruses also harbor additional modifications to serve as a vehicle for delivering therapeutic genes [72,74].

The functions of several HSV-1 genes could not be identified in tissue culture; therefore, these genes were considered non-essential; however, in animal models, these genes were found to have a role in disease induction and the virulence of the invading virions. This is controversial as, for example, HSV glycoprotein C (*gC*) is a non-essential gene with its roles, including binding to heparan sulfate proteoglycans (HSPGs) on the cell surface as the initial step of the infectious process. Still, *gB* also fulfills this role, and thus the function of *gC* is not essential, although the virus does replicate better with *gC* intact [32,75]. Also, *gC* binds to complement during infection in vivo. To note, non-essential for replication is defined as the role of that gene in infection of the virus in culture [76].

During recombinant virus generation for employment as an attenuated vector in vaccine development, the possibility of over-attenuating the virus could abrogate its usefulness. To lessen this likelihood, the progress of extra deletions has to be included and assessed for final and future over-attenuation. Attenuated HSV vectors have been assessed as live viral vaccines, gene therapy vectors to convey transgenes to the nervous system, and oncolytic viruses.

### 1.7. Bacterial Artificial Chromosomes (BACs)

BACs are artificial DNA constructs dependent on a functional F-plasmid employed for cloning huge DNA in *Escherichia coli* (*E. coli*) [77,78]. F-plasmids are used to synthesize BACs because they permit equal and even distribution of the plasmids and their viral inserts in multiplying bacteria. The large size of the herpesvirus genome represents an obstacle to successful ligation positioning of the BAC plasmid cassette within the viral genome, which occurs through homologous recombination following cell invasion. This happens because of the circularization of the linear double-stranded DNA viral genome during the viral replication process [16]. Isolated BAC DNA is then transfected into *E. coli*, which propagates the BAC and maintains the entire BAC when frozen at −80 °C. Transfection of the purified BAC DNA into eukaryotic cells enables the production of infectious virus particles [79]. The viral progeny after that produced by transfecting the kept viral genome in *E. coli* is mainly transfected through the BAC system into eukaryotic cells, creating recombinant viral genomes with the introduced foreign gene of interest in an expeditious, convenient, and feasible way.

## 2. Human Herpesviruses as Vectors (HSV, CMV, and VZV)

### 2.1. Herpes Simplex Viruses

Both attenuated and non-replicating HSV recombinants are promising viral vaccine vectors [32,80], and their applications in cancer gene therapy have exhibited satisfactory safety [68,81]. These promising criteria arise from several advantages of HSV. It can infect several tissues and species. It can also trigger durable immunity after several inoculation routes involving the mucosal application method [82]. It can accommodate large-size inserts of heterologous immunogenic foreign genes [83], which are considered advantageous characteristics for a vaccine vector. HSV-1 stimulates pro-inflammatory cytokines and type I interferons by triggering toll-like receptor 2 (TLR2) and activating TLR9, respectively [84,85,86,87]. In no case do vector genomes integrate into host chromosomes, consequently hindering the associated risk of insertional mutagenesis [33]. HSV vectors are immunogenic even in the presence of preexisting immunity [88].

For example, the vaccination of guinea pigs with VC2 HSV vaccine causes a transcriptional profile in their vaginal tissues that is indicative of Th17 and regulatory Th1 responses [89,90,91]. In addition, the non-neurotropic highly attenuated gK-deleted virus has been applied to induce durable protection against the genital infections of both virulent HSV-1 and HSV-2 [92]. Replication-defective HSV delivered intramuscularly showed antigen expression in muscle syncytia and improved combating pathogenic HSV-2 strains [93]. It elevated polarizing cytokines and chemotactic factors involving macrophage inflammatory protein-1*α* (MIP-1*α*) and interleukin-12p70 (IL-12p70), which were strongly linked to the magnitude of group-specific antigen (gag)-specific responses [94] and polyfunctional T cell responses to HIV-1 envelope (env); it strongly boosted the responses conferred to an adenovirus prime [95]. The human dendritic cells (DCs) transduced with HSV amplicons encoding HIV-1 gp120 elicited adaptive immune responses that conferred partial protection against HIV-1 challenge [96]. The extent of the amplicon-triggered immune response was strongly correlated with gag-level production in vitro and in vivo, which directly correlated to antigen expression [97].

**Figure 3:**
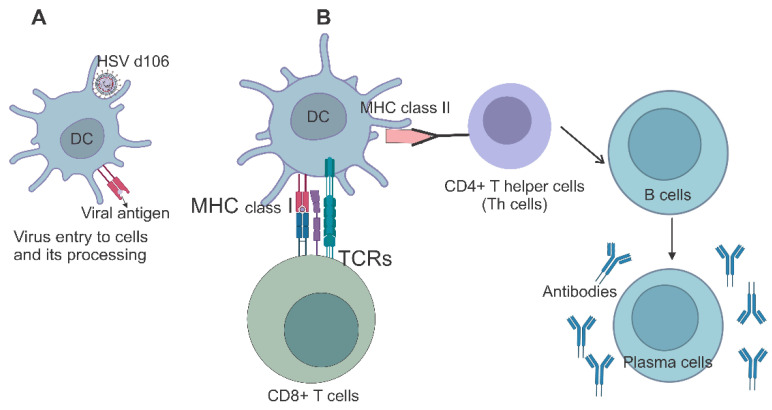
Humoral and cellular immune responses triggered by recombinant HSV d106: The recombinant viral HSV d106 vector enters the host cell through fusion with the plasma membrane or endocytosis, depending on the specific receptor interactions involved (**A**). Once inside the cell, the vector releases its genetic material, which consists of the coding sequences for the desired antigen(s). These genetic sequences are subsequently transcribed and translated by the machinery of the host cell. The recombinant viral vector primarily targets antigen-presenting cells (APCs), particularly dendritic cells (DCs) (**A**). DCs are proficient APCs that hold vital significance in the commencement of immune responses. The vector can invade DCs via receptor-mediated endocytosis or direct fusion with the plasma membrane. Post-infection, the recombinant viral vector incites both inherent and adaptive immunological reactions. The vector’s genetic content is identified by pattern recognition receptors (PRRs) situated in the cytoplasm or endosomes of the infected cells. This recognition triggers type I interferon (IFN) and pro-inflammatory cytokine synthesis, consequently triggering innate immune cell activation and the onset of adaptive immunity molecules in cellular immunity. CD8+ T cells, also called CTLs, recognize antigen/MHC complexes through their T cell receptors (TCRs) (**B**). Upon activation through this interaction, CTLs proliferate, differentiate into effector cells, and obtain cytotoxic functions. Subsequently, CTLs can detect and eradicate infected cells by displaying viral antigens on their surface, effectively controlling viral spread. For antibody development, infected DCs also present viral antigens via MHC class II molecules to CD4+ T helper cells (Th cells). Activated Th cells help B cells, stimulating their proliferation and differentiation into plasma cells. Plasma cells produce significant amounts of antibodies that target viral antigens. These antibodies can neutralize the virus by attaching themselves to its surface proteins, which prevent viral entrance into host cells and further encourage clearance by other immune mechanisms.

Several genes of HSV have essential roles in immune evasion, so their removal or inactivation enhances their properties concerning viral vector applications. Inactivation or removal of the IE genes *ICP4*, *ICP27*, and *ICP47* is anticipated to improve vaccine immunogenicity because the first two genes lead to the downregulation of major histocompatibility complex (MHC) class I in infected cells by decreasing antigenic presentation and also influence the transcription of host genes. *ICP47* is markedly involved in blocking TAP and, consequently, preventing the transport of peptides to the endoplasmic reticulum (ER) to be loaded onto MHC class I to be presented to immune cells [98]. In addition, *ICP22* has been recorded to inhibit MHC class II presentation [99]. *ICP4* and *ICP27* are essential IE genes; thus, one cannot take BACs containing these deleted genes unless one has a complementing eukaryotic cell line expressing those complementing genes in trans. Moreover, these two genes do not have any role in MHC-1 presentation; only *ICP47* does. The d106 strain as a second-generation HSV-1 replication-defective vaccine vector has shown high expression of the transgene and limited cytopathogenicity, ability to generate long-lasting immune responses, and induces maturation of dendritic cells in the lymph nodes [100,101]. The recombinant d106 contains deletions of *ICP22* and *ICP47* non-essential genes and deletions of the *ICP4* and *ICP27* essential genes, as Neal Deluca’s lab-engineered d106; once again, it needs a complementing cell line to grow d106 BAC viruses. HSV strains are generated as promising vaccine candidates against several microorganisms such as HSV infections [88,102], simian immunodeficiency virus (SIV) [103], HIV env [104], and HSV-1 [105]. HSV-2 ∆gD is also an ideal vaccine vector to stimulate the immune response against a vast plethora of pathogens. Their trial and efficacy are shown in Table 1. Here are two detailed examples of HSV as a vector vaccine for West Nile Virus (WNV) and equine herpesvirus type 1 (EHV-1). The first study includes the formulation of a vaccine against WNV. WNV, a type of flavivirus, has caused notable human illness in the United States [106], and at present, there is no approved vaccine for WNV. To construct an effective WNV vaccine, the authors used a replication-deficient HSV. This process involved the expression of WNV structural proteins, namely the premembrane (prM) and envelope (E), by employing optimized expression cassettes. With the intent to enhance the expression of WNV cassettes, the researchers conducted a variety of transfection studies. These studies identified the precise sequences for the optimal production of virus-like particles (VLP). They found that the 18 C-terminal residues of the C protein of the capsid served as an effective signal sequence for membrane insertion and VLP formation. The inclusion of these residues in the expression cassette resulted in high levels of E protein expression and VLP production. Based on these findings, the researchers constructed a recombinant HSV vector, d106-WNV, expressing the prM and E proteins fused to the C-terminal residues of the C protein. They then analyzed the kinetics of E protein expression and VLP production in infected Vero cells. The d106-WNV virus-infected cells exhibited high levels of E protein expression and secreted VLPs containing E protein. Electron microscopy confirmed the VLPs’ structure, which resembled genuine WNVs. Next, the researchers evaluated the immunogenicity of the d106-WNV recombinant vector in mice. The mice were immunized with three doses of the d106-WNV virus, and serum samples were collected at various time points to measure antibody responses. The results showed that immunization with d106-WNV elicited a specific anti-WNV IgG response in mice. Neutralization assays showed that immunized mice had WNV neutralizing antibodies in their serum. This research provides significant insights into strategic approaches for the development and enhancement of vaccine delivery systems targeted specifically at flaviviruses such as WNV. The use of a replication-defective HSV vector allowed for long-term expression of WNV proteins and the induction of antibody and CD8+ T cell responses (Figure 3). The findings of this research demonstrate the feasibility of employing HSV vectors as a predominant approach to immunization when employed alongside other vaccination techniques. This combination approach can lead to a stronger immune response and better protection against WNV infection.

Another study evaluated the potential use of the live attenuated HSV-1 VC2 vaccine strain as an avenue for constructing a vaccine against EHV-1 infection. By integrating the EHV-1 *gD* gene into the VC2 vector, a recombinant virus, VC2–EHV-1–gD, was synthesized [107]. The VC2 vaccine strain was selected due to its incapacity to enter neurons and establish latency in mice. The vaccine was administered to mice through intra-muscular injection. The results showed that vaccination with both VC2–EHV-1–gD induced the production of neutralizing antibodies, which were significantly higher than non-vaccinated and VC2-vaccinated animals. The VC2–EHV-1–gD vaccine also stimulated robust IgG1 and IgG2a antibody responses, indicating both Th1 and Th2 immune responses. In addition to humoral responses, vaccination with VC2–EHV-1–gD also stimulated strong cellular immune responses. The administration of the vaccine triggered a rise in interferon and tumor necrosis-factor-positive CD4+ and CD8+ T cells, signifying the elicitation of cell-mediated immune processes. Moreover, the histopathological analysis demonstrated reduced inflammation levels in the lung tissues of mice vaccinated with VC2–EHV-1–gD compared to those vaccinated with VC2 alone or non-vaccinated mice after the EHV-1 challenge. This implies a potential protective effect of the vaccine against EHV-1-induced lung pathology. The study illustrated that the HSV-1 VC2 vaccine strain expressing EHV-1 gD can effectively stimulate both humoral and cellular immune responses in mice.

**Table 1:** Experimental HSV-based vaccine vectors. SIV, simian immunodeficiency virus; HIV, human immunodeficiency virus; IAV, influenza A virus; WNV, West Nile virus, HSV, herpes simplex virus; FMDV, foot and mouth disease virus; EHV-1, equine herpesvirus type 1.

Viral Infection	Targeted Antigens	Experimental Immunization in	Immune Response	Refs.
SIV	Env and Nef	Rhesus macaques	Specific humoral immune response, partial protection	[103]
Rotavirus-	VP2, VP6, and VP7	Mice	Specific humoral immune response, partial protection	[108]
HIV	gp120	Mice	Strong humoral and cell-mediated immune responses	[104]
IAV	Modified IAV hemagglutinin (HA)	Mice and guinea pigs		[109]
WNV	Env and prM	Mice	Strong humoral and cell-mediated immune	[106]
HSV-1	gD	Mice	Strong humoral and cell-mediated immune responses	
FMDV	P12A3C	Mice	Specific humoral response and partial protection	[110]
FMDV	P12A3C	Mice	75% protection from challenge (Ad/herpes vector, prime-boost)	[111]
HIV-1	HIV-1 gp160	Adult female BALB/c mice	Antibody responses were measured by ELISA and the induced cell immune responses were measured by intracellular cytokine staining	[112]
Malaria	EXP1, UIS3, TMP21, VP26	BALB/c mice	Potent cellular and humoral immune responses. Protected against challenge in mice	[113]
SARS-CoV-1	spike protein	Balb/C and C57BL/6J mice strains	Antibodies and expression of cytokines were induced	[114]
EHV-1	glycoprotein D	Female BALB/c mice	Strong antiviral humoral and cellular immune responses	[107]

### 2.2. Human Cytomegalovirus

Cytomegaloviruses (CMVs) are ubiquitous beta herpesviruses that can establish lifelong infections with a low-level persistence within the host. Infection with CMV occurs regularly without symptoms and typically occurs in early childhood or during adolescence. CMV infections can be particularly severe prenatally and in those with compromised immune systems [115,116]. Despite their capacity to provoke an immunogenic response, these viruses can still cause reinfection in patients who have already been infected.

As large DNA viruses, CMVs possess several features, making them attractive vaccine vector candidates. These characteristics involve the large genome that accommodates foreign gene insertion [117,118,119], species specificity [120,121,122], and persistent triggering of the immune response during the persistent and latent state. Also, infections are not only host-specific but cell-type-specific. Thus, the human CMV (HCMV) only infects and replicates in human monocytes [123,124,125,126].

The CD8 T cell responses to some CMV antigens display “memory inflation,” which means that the responding memory population is maintained at a high level or raised slowly over time instead of a smaller memory population maintenance or rapid contraction following the initial response post-infection. Importantly, these responding cells can enter the non-lymphoid tissues [127,128,129,130]. These properties favor the possibility of using CMVs as vaccine vector platforms [122,130,131]. BAC technology has facilitated the construction of recombinant CMVs (rCMVs) expressing antigens under several different promoters. Additionally, BAC technology has enabled the exploration of the role of CMV genes and the elimination of those that disrupt the body’s immune system, thus augmenting their protective prowess [132,133,134]. CMV recombinants have been constructed against many viral diseases, such as SIV, Ebola virus, and HSV (shown in Table 2) [135,136]. According to several reports, a CMV-based vector vaccine prepared in rhesus macaque monkeys could protect macaques against SIV challenges by the induction of CD8 T cells restricted by MHC class II, in addition to a large collection of various T cells specific for several epitopes of the target antigens [137].

A recombinant murine CMV vector was constructed in such a way that it deleted several viral genes, but the viral genes that interfere with MHC class I expression were deleted from the vector [138,139,140,141]. The murine CMV85A (MCMV85A) recombinant vector induced protective immunity against *M. tuberculosis* compared to the control virus, empty MCMV (eMCMV), which produced a weaker effect [142], as shown in Table 2.

**Table 2:** Experimental CMV-based vaccine vectors. EBOV, Ebolavirus.

Disease, Bacterial or Viral Infection	Targeted Antigens	Experimental Immunization in	Immune Response	Refs.
EBOV	NP CD8+ T cell epitope	Mice	Long-lasting CD8+ T cell response, complete protection	[125]
*Clostridium tetani*	Tetanus toxin fragment C	Mice	Specific humoral response	[143]
Cancer	Mouse tyrosinase-related protein 2	Mice	Prolonged patient survival	[144]

### 2.3. Varizella Zoster Virus

VZV is an HHV that causes chickenpox and shingles, as well as viremia and different stages of skin lesions [145,146,147]. The vOka strain of the varicella virus, a live attenuated form, was created through the adaptation of the pOka parent strain [148,149,150], which is an attenuated Japanese clinical isolate produced in semi-permissive guinea pig embryo fibroblasts [151]. Because of its efficiency and few side effects, it is the sole globally utilized vaccine strain, particularly for immunizing children [57,152,153,154]. vOka possesses a genome comprising double strands of DNA with approximately 125 kbp, encompassing at least 71 ORFs. Another advantage of vOka is that its large genome contains numerous non-essential genes not needed for viral replication that make the foreign genes be readily incorporated into the viral genome as *ORF13* gene [155,156,157]. The VZV Oka parental strain and a vOka BAC vector were efficiently reconstituted to their recombinant viruses by Nagaike et al. [158] and Yoshii et al. [159]. Thus, vOka has been utilized to express hepatitis B surface antigen [160], HSV-2 [161], RSV [162], EBV membrane glycoprotein (gp350/220) [163], mumps virus H–N and/or F protein [164,165,166], HIV env [167], and HSV-2 glycoproteins B and D [168]. It was also found that the rvOk strain harboring the mumps virus gene or respiratory syncytial virus (RSV) A-F or RSV B-F triggered antibodies in immunized guinea pigs against the specific proteins [162,166]. Varicella vaccine strain, vOka, exhibited several advantages, such as inducing long-lasting immunity and its safety and efficacy, making it an attractive live vector [162,168,169] (Table 3). Even though vaccine development with herpesviruses has been successful, difficulties in engineering vaccines and producing high-titer preparations of cell-free viruses remain, with the most obstacles being associated with VZV-based vaccines.

## 3. Molecular Mechanisms and Pathways Used Herpesviruses as Vaccine Vectors

Herpesviruses have garnered significant attention as vaccine vectors due to their unique molecular mechanisms and pathways. These viruses can be genetically engineered to deliver and express foreign antigens, thereby stimulating robust immune responses. Understanding the intricate molecular mechanisms and intricate pathways implicated in harnessing the power of herpesviruses as vehicles of vaccines is of utmost importance in order to fully unleash their potential in crafting potent vaccines against a myriad of infectious diseases. The following are the molecular mechanisms and pathways that support herpesviruses as vaccine vectors:

### 3.1. Immune Response Stimulation

One of the main molecular mechanisms of herpesvirus-based vaccine vectors is their capacity to incite robust immune responses. They can trigger the induction of both humoral and cellular immune responses. These viruses stimulate the induction of neutralizing antibodies to bind viral antigens, preventing infection and aiding in viral clearance [107,170]. They additionally induce a potent cellular immune response by activating CD4+ and CD8+ T cells. CD4+ T cells promote antibodies and activate and direct cytotoxic CD8+ T cells to clear virus-infected cells [107,170]. The synergistic effect of these immune responses greatly amplifies the strength of herpesvirus-based vaccines. This collaboration between immune responses effectively augments the power of herpesvirus-based vaccines.

### 3.2. Genetic Manipulation and Antigen Presentation

Genetic manipulation is a fundamental aspect of utilizing herpesviruses as vaccine vectors. This process involves modifying the viral genome to optimize safety, immunogenicity, and antigen expression. Genes associated with virulence or latency can be deleted to attenuate the virus, reducing the disease risk [100,101]. On the contrary, the introduction of genes that can enhance the immune response, such as immune modulatory cytokines or co-stimulatory molecules, enhances the effectiveness of the vaccine. Herpesviruses proficiently transport foreign antigens to host cells, processed and presented on MHC molecules [122]. This antigen presentation triggers an immune response, contributing to the strong immune response generated by herpesvirus-based vaccines.

### 3.3. Persistence and Longevity of Immunity

Another noteworthy molecular mechanism of herpesvirus-based vaccine vectors is their ability to establish persistent infections. After vaccination, herpesviruses can enter host cells and establish latent infections [171,172,173,174]. During latency, viral gene expression is limited, but the viral genome persists within infected cells. Periodic reactivation from latency leads to antigen expression, further boosting immune responses and contributing to the longevity of immunity provided by these vaccines [171,172,173,174]. The continuous antigen exposure resulting from latent infections helps maintain a robust and long-lasting immune response, enhancing the effectiveness of herpesvirus-based vaccines.

### 3.4. Immune Evasion Strategies

Herpesviruses have evolved sophisticated immune evasion strategies that enhance their effectiveness as vaccine vectors. These strategies enable herpesviruses to evade host immune responses, ensuring prolonged antigen expression and immune stimulation [175,176,177]. For instance, genes encoded by herpesviruses counteract host antiviral defenses, modulate antigen presentation pathways, and inhibit apoptosis. These immune evasion mechanisms help sustain prolonged expression of viral antigens, leading to a sustained and robust immune response induced by the vaccine vector.

## 4. Immune Responses and the Issue of Preexisting Immunity

The multifaceted and complex immune responses provoked by vector vaccines are those directed against the heterologous transgene, the vector itself, and the innate immune responses induced by the vector. These responses collectively influence the transgene-specific immune responses. The amount of transgenic antigen expressed and the cell type in which the vaccine vector replicates determine the magnitude and nature of the immune response. For instance, a higher level of transgenic antigen expression yields a potentially stronger immune response. Additionally, the targeted host cells can affect immune responses as various types of cells present antigens and trigger immune responses differently. Thus, understanding the interplay between these factors is critical to optimizing the efficacy of vectored vaccines. Herpesviruses, due to their ability to establish persistent infections, can induce lifelong immunity. However, this characteristic also presents a significant challenge when these viruses are used as vectors for gene therapy or vaccines in the human population [171,178,179]. Preexisting immunity from natural infections can lead to the continuous production of virus-neutralizing antibodies. These antibodies can significantly limit the effectiveness of vector vaccines by reducing the uptake of vaccine vectors by cells, including antigen-presenting cells [171,178,179,180]. This decrease in uptake can result in reduced expression of the transgene product, which then impacts the particular immune response triggered by the transgene. Neutralizing antibodies (nAbs) present a major hindrance to the practical use of vector vaccines in natural hosts. Nonetheless, various methods can be utilized to overcome these obstacles. One approach is to increase the vector dose, which can potentially enhance the immune response against the transgene. Another strategy is to use different species of herpesviruses that are not naturally hosted by humans, thereby avoiding preexisting immunity. Prime-boost regimens, which involve sequential administration of the same or different vaccines, can also be used to enhance adaptive immune responses to the transferred gene product while reducing the induction of antibody responses to the herpesvirus antigens. This strategy allows the vaccine to be more effective since the immune response is focused on the transgene. Additionally, combining herpesvirus vectors with other vectors, like adeno- or poxviruses, or microsphere encapsulation or polyethylene glycol (PEG) coating, can lessen vector neutralization. These methods can mask the vector from the immune system, enhance its capacity to deliver the transgene, and induce an effective immune response. In conclusion, while vector vaccines present significant potential for the prevention and treatment of various diseases, their effectiveness can be influenced by various factors. Understanding and tackling these factors is of vital importance to successfully developing and deploying these vaccines.

## 5. Discussion

An in-depth understanding of the molecular and immunological mechanisms that underlie the pathogenesis of herpesviruses will lead to more advances in vaccine development. Virus attenuations, which can be easily achieved by removing some virulence genes without affecting virus replication or antigenicity by codon deoptimization, clustered regularly interspaced short palindromic repeats/CRISPR-associated protein (CRISPR-Cas) techniques, or mutagenesis techniques, represent a hot spot in the development of vaccine vectors. Historically, the attenuation of viruses has been achieved via passaging in an unsusceptible host, transformed cell lines, or an unusual route of administration. This traditional attenuation may affect the DNA viruses, leading to the loss of some genes or parts of genes that have an impact on triggering immune response or affected innate immunity pathways, including interferon, cytokines, and others or the adaptive immunity by affecting major histocompatibility classes for antigen processing [32,181]. One of the aims to enhance the diverse types of immune responses is to add some adjuvants. In addition, knockout or removal of the genes responsible for immunoevasion will strengthen the host immune response for the inserted gene of interest. Still, the induced anti-vector immunity may affect reusing them in the individual. Even in the face of both innate and adaptive immune responses, herpesvirus vectors can be used in repeat administration, at times in the presence of high doses of anti-HSV nAbs [182,183,184,185]. These represent just a few of the recorded papers covering the re-administration of HSV vectors where the response is seen after re-dosing was greater than that before re-administration, suggesting the ability of the reapplied vector to avoid neutralizing antibodies (NAs) to the virus, or even the therapeutic gene, and, in the end, produced an even more robust therapeutic effect. This fits in with the fact that patients were experiencing HSV infections where viruses reactivate infrequently or that these patients may become reinfected with the virus later. Rational attempts to enhance immunogenicity by removing one or more genes are an interesting topic. Several issues can be considered to enhance the immunogenicity, including rational design of vectors, delivering genes to specific cells, combating preexisting immunity, a prime-boost regime, and using different strong promoters, adding some immunomodulatory cytokines or chemokines such as IFN-γ, interleukins, and suitable codon optimization.

The kind of foreign gene/s to use, the way to introduce it to the host, and the proper insertion location in the herpesvirus genome will generate the different needed immune responses to combat various infections. For antigen processing and memory inflation, it is necessary to know that the promoter implemented, the site of inserted epitopes, and its avidity must be considered. For clinical use trials, it is essential to assess the vaccine dosage and administration route as they impact the kinetics and strength of the immune response. Yet, more insights into antigen processing mechanisms and their presentation by MHC molecules to T lymphocytes are needed to understand how they may alter the inflationary response to any introduced foreign genes. Furthermore, additional information about the specific mechanisms for protecting the vaccine candidates is necessary to improve their design, together with a better understanding of the durable outcome of latent characteristics of natural immunity and its interactions with the preferred adaptive immunity. The greatest obstacle regarding some members of herpesviruses is the design of herpesvirus candidates that are safe and non-pathogenic for clinical use in immunocompromised individuals, particularly to avoid congenital infection and retain immunogenicity. Future efforts should be made toward its translation to clinics due to its species-specific proclivities and using animal herpesviruses for human use and vice versa.

## 6. Challenges, Opportunities, and Future Directions

The remarkable capability inherent in human herpesviruses to maintain a persistent latent infection in the human body and subsequently to reactivate their activity in the later stages of life provides them with an appealing attraction as potential candidates for use as vehicles for vaccine development. These versatile agents can be finely engineered to express antigens from a diverse array of pathogenic sources, thereby eliciting a robust and protective shield of immunity. However, using these viruses as vaccine vectors poses challenges. One concern is their potential pathogenicity and establishing latent infections that could reactivate. To overcome these obstacles, ongoing research is focusing on using attenuated viruses that are no longer pathogenic or genetically engineered viruses with essential pathogenic genes removed. Effective delivery systems for herpesvirus vectors are also being explored, such as intranasal or intravenous administration, to overcome the infeasibility of direct injection of the vector into the target tissue for certain applications, such as gene therapy for CNS disorders. Immune responses directed against these vectors due to preexisting immunity can restrict their effectiveness; therefore, approaches to override this immune response involve the use of immunomodulating agents and the development of vectors that can evade the immune system. Additionally, the development of an effective immune response against the desired antigen is crucial, and novel strategies like adjuvants and immunogenic components are being explored to overcome the sophisticated immune evasion mechanisms.

Apart from vaccine development, human herpesviruses have potential for gene therapy applications that employ genetic material to address and potentially cure diseases; however, there are still challenges to address for improved safety and efficacy. A key challenge is delivering genes to specific cell types and minimizing unwanted effects. Advances in viral vector engineering and genome editing technologies offer solutions. Regulating transgene expression is also essential to prevent toxicity and optimize therapeutic outcomes. Inducible and cell-specific promoters and regulatory elements show promise in achieving precise control. The future lies in addressing these challenges through research advancements in viral vector engineering, immunology, and gene editing technologies. Combination therapies and next-generation herpesvirus vectors with enhanced immunogenicity and safety profiles will expand such possibilities. Furthermore, studies on nonhuman primates and other mammals will provide invaluable perspectives on the effects of herpesvirus vectors.

Another future direction is engineering herpesvirus vectors to target specific cell types. Tropism-modified vectors are being designed through mutagenesis of viral glycoproteins or incorporation of antibodies. This targeting strategy enhances transgene expression in desired cell populations, improving safety and efficacy. Rational mutagenesis modifies viral envelope proteins to alter receptor binding specificity for specific cell types. Incorporating single-chain antibodies into the viral envelope redirects the vector toward cells expressing the target antigen. Targeting specific cell types allows precise delivery, enhances efficiency, and improves safety. Further research is needed to evaluate herpesvirus vectors in large animal models that mimic human physiology and immunology. This will provide crucial preclinical data for regulatory approval. Mice studies have been informative but do not fully replicate natural herpesvirus infections in humans.

## 7. Conclusions

Outbreaks of infectious diseases and their ongoing risk have driven the progress of research for designing novel, quickly adaptable, and safe vaccines. Herpesviruses boast a number of advantageous and unique traits, which make them ideal tools for producing efficient and effective vaccines to combat pathogenic agents. Herpesviruses are exhibiting substantial potential as antigen carriers for many pathogens, including FMD and HIV, and hopefully will be used for the COVID-19 pandemic. Nevertheless, despite their potential, there are certain drawbacks, such as limited studies and safety concerns associated with using herpesvirus vectors to create vaccines. A thorough comprehension of herpesvirus biology is essential to overcome any lingering limits. Recent significant advancements in recombinant genetic engineering have dramatically driven the development of vector vaccines. With further understanding and innovative research, we may yet unlock the full potential of herpesviruses for effective vaccine production.

## Figures and Tables

**Table 3:** Experimental VZV-based vaccine vectors. HIV, human immunodeficiency virus; MuV, mumps virus; HBV, hepatitis B virus; HSV, herpes simplex virus.

Viral Infection	Targeted Antigens	Experimental Immunization in	Immune Response	Refs.
HIV	Env	Guinea pigs	Strong humoral and cell-mediated immune responses	[167]
MuV	Hemagglutinin-neuraminidase	Guinea pigs	Specific humoral response	[166]
HBV	preS2 and the complete S region	Guinea pigs	Specific humoral response	[160]
HSV-2	gB and gD	Guinea pigs	Significant protection (high antibody titers, reduced lesions, and reduced mortalities)	[168]
HSV-2	gD	Guinea pigs	Significant protection (high antibody titers, reduced lesions, and reduced mortalities)	[161]

## Data Availability

No new data were created or analyzed in this study. Data sharing is not applicable to this article.
